# Most Plant-Based Milk Alternatives in the USDA Branded Food Products Database Do Not Meet Proposed Nutrient Standards or Score Well on Nutrient Density Metrics

**DOI:** 10.3390/nu14224767

**Published:** 2022-11-11

**Authors:** Adam Drewnowski

**Affiliations:** Center for Public Health Nutrition, University of Washington, P.O. Box 353410, Seattle, WA 98195, USA; adamdrew@uw.edu

**Keywords:** plant-based beverages, milk alternatives, Nutrient Rich Food index, Choices International, Nutri-Score, branded food products database, ingredients, Nestlé nutrient standards

## Abstract

Plant-based milk alternatives and plant-based waters are of variable nutritional value. The present objective was to assess nutrient density of all plant-based beverages in the US Department of Agriculture Branded Food Products Database and determine whether plant-based milk alternatives met the proposed nutrient standards. Plant-based milk alternatives (*n* = 1042) were identified as almond, soy, coconut, cashew, other tree nut, flax/hemp, pea, and oat, quinoa, and rice products. Plant-based waters (*n* = 550) were coconut, aloe, tree, fruit, and plain. Machine searches of ingredient lists identified products with added sugars, salt, vitamins, and minerals. Plant-based milk alternatives were tested for compliance with previously developed nutrient standards. The Nutrient Rich Food Index (NRF5.3), two versions of Nutri-Score, and Choices International were the nutrient density metrics. Plant-based milk alternatives had mean energy density of 49 kcal/100 g, were low in protein (~1.1 g/100 g), often contained added sugars and salt, and tended to be fortified with calcium, vitamin A, vitamin D, and vitamin B12. Only 117 milk alternatives (11.2%) met nutrient standards and only 80 (7.7%) met the more stringent “best of class” standards for ≥2.8 g/100 g protein and <3.1 g/100 g added sugars. The latter were mostly soy milks. Nutri-Score grades varied depending on whether the beverages were treated as beverages or as solid foods, as is currently required. The highest NRF5.3 scores were given to soy, almond, and tree nut milk alternatives. Plant-based waters had low energy density (~23 kcal/100 g), contained added sugars (4.6 g/100 g), and some had added vitamin C. Applying nutrient standards to plant-based milk alternatives can aid new product development, promote more transparent labeling, and inform potential regulatory actions. Guidance on minimum protein content, maximum recommended amounts of fat, added sugars, and sodium, and consistent fortification patterns would be of value to regulatory agencies and to the food industry.

## 1. Introduction

Plant-based beverages, including plant-based (PB) milk alternatives, are a rapidly growing market segment in the US and worldwide. PB milk alternatives include those made from soy, almonds, oats, and from other legumes (pea), grains (quinoa), or nuts (macadamia) [1,2]. PB milk alternatives are often viewed as equivalent to milk in nutritional value [3,4]. However, their energy and nutrient content can be highly variable, depending on product type and brand [5,6].

Low-fat (1%) milk typically contains 3.3 g/100 g of protein, 0.6 g/100 g of saturated fat, and about 5.0 g/100 g of naturally occurring lactose sugar. In the US, both milk and PB milk alternatives are fortified with vitamin A and vitamin D [7]. Ensuring that PB milk alternatives provide adequate nutritional value that is not inferior to milk has become an issue of public health concern [8,9]. The US Food and Drug Administration (FDA) is in the process of developing Guidance for Industry on the labeling of PB milk alternatives [10] and has sought comment on the use of dairy terms for non-dairy beverages and foods [11]. A set of proposed nutrient standards for PB milks was recently developed to guide product formulation and for potential use by regulatory agencies [12]. At that time, only <5% of PB milk alternatives in the USDA Branded Food Products Database (BFPDB) met the proposed standards for protein, saturated fat, and added sugars, and for those vitamins and minerals that are associated with milk [12].

The 2021 version of the BFPDB, available on the USDA FoodData Central, provides energy and selected nutrient values for over 300,000 foods and beverages, together with their declared ingredients [13]. PB milk alternatives and PB waters were already identified by the USDA. This study used machine searches of product names and ingredients to identify the main plant sources of PB milk alternatives (nuts, grains, legumes); the main forms of added sugars and sodium; and the chemical names of added vitamins and minerals. The goal was to determine how many PB milk alternatives met the proposed nutrient standards [12] and to assess their overall nutritional value using well-established nutrient density metrics.

At this time, there are no proposed nutrient standards for PB waters, other than the general agreement that these products too ought to offer some nutritional value. PB waters are not intended as milk alternatives; rather they are alternatives to sugar-sweetened beverages that can contain up to 10 g of added sugars per 100 g product. A secondary goal was to better characterize sugar content and fortification patterns of PB waters. Three nutrient profiling models: Nutrient Rich Food Index [14]; Nutri-Score [15], and Choices International [16] were used to assess the products’ nutritional values.

## 2. Materials and Methods

### 2.1. The USDA Branded Food Products Database (BFPDB)

The USDA Branded Food Products Database (BFPDB) is publicly available and can be downloaded from US Agricultural Data Commons [13]. Product category assignments into PB milk alternatives (*n* = 1065) and PB waters (*n* = 558) were made by the USDA. The BFPDB provides product long name, manufacturer name, energy content (kcal/100 g), and nutrient values per 100 g for most of the nutrients on the Nutrition Facts Panel, along with a list of product ingredients as declared by the manufacturers on the food label.

Beverages identified by the USDA as PB milk alternatives did not necessarily have the word “milk” in the product name. Included in the category were PB beverages with alternative spellings, products with adjectival names (milked hazelnuts), PB drink or beverage blends, and PB beverages flavored with coffee, fruit, and other flavors. Analyses followed the USDA categorization scheme.

The US Code of Federal Regulation Section 101.4, Title 21 [7] requires ingredients to be listed in order of predominance, with those used in the greatest amount to be listed first. The plant origin of PB beverages was identified using machine searches of product long names and ingredients. Search terms included almond, cashew, coconut, flax, hemp, macadamia, walnut, peanut, pistachio, pecan, rice, quinoa, oat, pea, soy, sunflower, and sesame. Search terms specific to PB waters included cactus, aloe, maple, birch, and a variety of fruit. In blended products (e.g., almond and coconut or pea and cashew), the plant source listed first on the ingredient list was decisive for category assignments. For PB beverages with names such as “golden milk” that provided no hint of plant source, the plant source listed first on the ingredient list was also decisive. This approach can be used in other markets. While not required in the US, a quantitative declaration of ingredients (QUID) is required in the European Union when the ingredient (or a category) is part of the food name [17].

**Figure 1:**
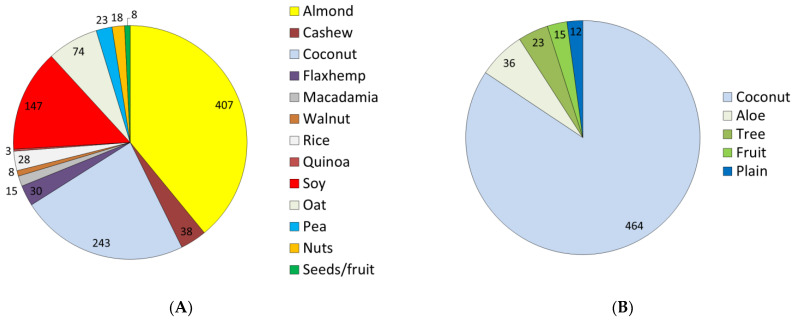
PB milk alternatives (**A**) and PB waters (**B**) by main ingredient (**B**).

Excluded from the analytical sample were PB beverages with energy density that was either missing or reported as >250 kcal/100 g, a value more consistent with solid foods. The 1042 PB milk alternatives were coded as almond (*n* = 407), cashew (*n* = 38), coconut (*n* = 243), flax/hemp (*n* = 30), macadamia (*n* = 15), walnut (*n* = 8), rice (*n* = 28), quinoa (*n* = 3), soy (*n* = 147), oat (*n* = 74), pea (*n* = 23), tree nut (*n* = 18), and fruits and seeds (*n* = 8). The 550 PB waters were coded as coconut (*n* = 464), aloe (*n* = 36), birch or maple (*n* = 23), fruit (*n* = 15) and flavored (*n* = 12). PB beverage distribution by main ingredient is shown in Figure 1.

### 2.2. Imputed Nutrient Values

Even though the USDA Branded Food Products Database had undergone quality control, there were some discrepancies and missing values. Missing values for vitamin A, vitamin D, and vitamin B12 were imputed based on the presence of the vitamin on the ingredient list and median amounts for product type. Missing values for products with no vitamin declaration were set at 0. Data on added sugars were not available.

### 2.3. Nutrient Profiling Models

The Nutrient Rich Food Index (NRF5.3) was based on five nutrients to encourage and three nutrients to limit. Each nutrient was expressed as a percentage of daily values (%DVs), calculated per 100 kcal and capped at 100%. The NRF5.3 formula is NRF5.3 = NR5–LIM, where NR5 is the positive NR5 sub-score and LIM (an abbreviation for nutrients to limit) is the negative sub-score [14]. Nutrients and reference amounts for the NR5 sub-score were: protein (50 g), vitamin A (2666 IU), vitamin D (800 IU), calcium (1300 mg), and vitamin B12 (2.4 mcg). Nutrients and reference amounts for the negative LIM sub-score were saturated fat (20 g), total sugars (90 g), and sodium (2300 mg). Beverages with energy density <10 kcal/100 g were eliminated to avoid dividing by zero.

The Nutri-Score [15] does not consider PB milk alternatives to be beverages; following current regulations, all PB beverages are to be treated as solid foods [18]. Two versions of Nutri-Score were, therefore, used; one for beverages and one for solid foods. For solid foods, negative “A” points are based on calories, saturated fat, total sugars, and sodium. The initial thresholds are >335 kJ/100 g for energy and >4.5 g/100 g for total sugars. The positive “C” points are based on protein and fiber. When total “A” points <11. then theNutri-Score is A–C. However, when total “A” points >11, the food must be >80% by weight fruit, vegetable, legume, or nut for protein points to be included. For those items, the Nutri-Score is “A” points minus the fiber points only.

For beverages, the energy and total sugar thresholds are much more stringent. The initial threshold for energy is >30 kJ/100 g and for total sugars >1.5 g/100 g. Only plain water is considered as having the “highest nutritional value”. Nutri-Score points convert into color-coded letter grades. For solid foods, final score of <−1 points becomes A; 0 to 2 points become B; 3 to 10 points become C; 11 to 18 points become D, and point scores >19 become E. For beverages, water is A; minimum to 1 point is B; 2 to 5 points is C, 6 to 9 points is D, and >10 points is E.

The Choices International [16] score has a separate category of non-dairy milk alternatives. Here, the scoring criteria are based on saturated fat <1.1 g/100 g; sodium <100 mg/100 g; total sugars <5.0 g/100 g; and beverage energy <40 kcal/100 g. Other than energy, those criteria were like the proposed standards. All criteria need to be satisfied for a product to receive a favorable “pass” rating.

### 2.4. Ingredient Searches for Added Sugars, Salt, Vitamins, and Minerals

Added sugars from syrups and honey; concentrated or evaporated cane syrup and cane juice; corn; maple; date; rice or brown rice syrup; coconut sugar; fruit juice concentrate; and date juice are treated as added sugars by the FDA [19]. Accordingly, ingredient lists were searched for sugar, cane sugar, pure cane sugar, dried cane syrup, evaporated cane syrup, evaporated cane juice, evaporated cane juice syrup, brown rice syrup, honey, organic maple syrup, date syrup, and organic dates.

Search terms for added sodium were salt, sea salt, Himalayan pink salt, and sodium. Search terms for added calcium were calcium carbonate, tricalcium phosphate, and calcium lactate. Searches were conducted for vitamin A palmitate and acetate, ergocalciferol (vitamin D), alpha-tocopherol (vitamin E), ascorbic acid or ascorbate (vitamin C), vitamin B12, zinc oxide, sulfate, and gluconate. Some products and ingredient lists are shown in Table 1.

### 2.5. Proposed Nutrient Standards for PB Milk Alternatives

Previously proposed nutrient standards for PB milk alternatives [10] were based on desired energy per serving, minimum protein content, and proposed limits on saturated fat, total and added sugars, and sodium. Included were minimum fortification levels for those vitamins and minerals that are commonly associated with milk. The proposed standards per 100 g for children and adults are shown in Table 2.

### 2.6. Statistical Analyses

Tests for significant differences in mean energy density, nutrient content, and nutrient density of PB products by beverage type and by main ingredient (Table 3 and Table 4) were based on one-way ANOVAS that were followed in some cases by post hoc tests adjusted using the Bonferroni correction for multiple comparisons. Correlations between nutrient density scores were Spearman correlations. Differences between Nutri-Score values for beverages and for solid foods were tested using paired t-tests. Significance level was set at *p* < 0.01. The IBM SPSS Statistical Package v16.0 (International Business Machines, Armonk, NY, USA) was used for statistical tests.

## 3. Results

### 3.1. Nutrient Composition of PB Milk Alternatives and PB Waters

Mean energy and nutrient content of PB products varied by product type. Table 3 shows that mean energy density was 49 kcal/100 g, ranging from 27 kcal/100 g (almond) to 95 kcal/100 g (coconut). Energy density of coconut milk was significantly higher than for all other PB products (*p* < 0.001), except quinoa. Mean protein content was 1.1 g/100 g or below, well below that of dairy milk. Only pea and soy beverages contained protein at levels comparable to milk: pea products contained a mean of 3.46 g/100 g and soy contained 2.82 g/100 g. The main effect for protein content was significant (*p* < 0.001); post hoc tests confirmed that both soy and pea protein levels were significantly higher compared to all the other PB milk alternatives. The protein in several PB milk alternatives came from pea protein concentrates or isolates, sometimes together with rice proteins.

Mean content of total sugars was low (2.80 g/100 g), ranging from 1.29 g/100 g (macadamia) to 4.15 g/100 g (oat) and 5.05 g/100 g (rice). Total fat in coconut milk was significantly higher than for all the other PB milk alternatives (*p* < 0.001). Saturated fat was generally very low (<0.42 g/100 g), other than for coconut milks (7.43 g/100 g), which were significantly above every other PB product (*p* < 0.001). Mean calcium content was 97.6 mg/100 g, ranging from a low of 23.7 mg/100 g (coconut) to 180.0 mg/100 g (pea). Mean sodium content was generally below 60 mg/100 g.

PB waters had low energy density (23 kcal/100 g) and contained no protein, no saturated fat, and very little sodium. Total sugars content was 4.6 g/100 g, well below that of most sugar-sweetened beverages, which typically contain about 10 g/100 g of added sugar. 

### 3.2. Added Sugars, Sodium, Vitamins and Minerals

**Figure 2:**
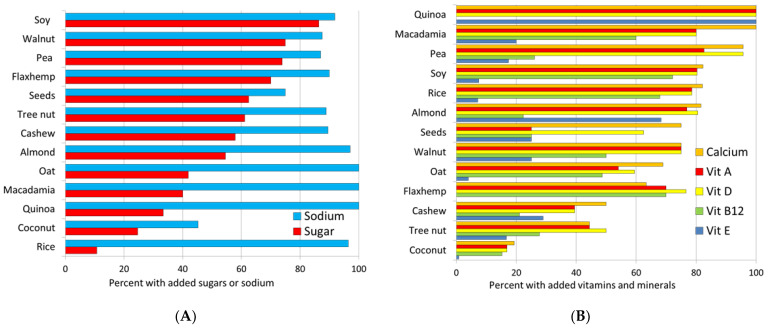
Percent of PB milk alternatives with added sugars and sodium (**A**), and percent of PB milk alternatives fortified with micronutrients (**B**). PB are classified by main ingredient.

About half of the PB milk alternatives contained added sugars (n = 51.0%). Figure 2A shows that PB milk alternatives from soy, walnut, pea, and flax/hemp were most likely to be sweetened; rice and coconut beverages were least likely to be sweetened. Most PB milk alternatives contained added sodium, either as salt or as sodium salts of added nutrients. The mean sodium content was only 47 mg/100 g, less for PB waters (20 mg/100 g).

For PB milk alternatives, fortification with calcium was most common (*n* = 672), followed by vitamin D (*n* = 648), vitamin A (*n* = 620), vitamin B12 (*n* = 344), vitamin E (*n* = 210), and zinc (*n* = 110), Specific fortification patterns varied by product type. For example, all quinoa products were fortified with calcium as well as vitamins A, D, and E, but not vitamin B12. Soy products were fortified with calcium and vitamins A, D and B12, but not vitamin E.

Figure 2B shows that PB milk alternatives made from quinoa, macadamia, pea, soy, and almonds were most likely to be fortified with calcium and vitamins. Coconut and cashew products were least likely to be fortified. The ingredient list pointed to the use of a fortificant mix composed of calcium, vitamin A, and vitamin D. The most frequent ingredient was tricalcium phosphate.

About 25% of PB waters were fortified with vitamin C (*n* = 124), fewer with calcium (aloe), and on rare occasions with vitamin A, vitamin E, or vitamin B12.

### 3.3. Nutrient Standards for PB Milk Alternatives

**Figure 3:**
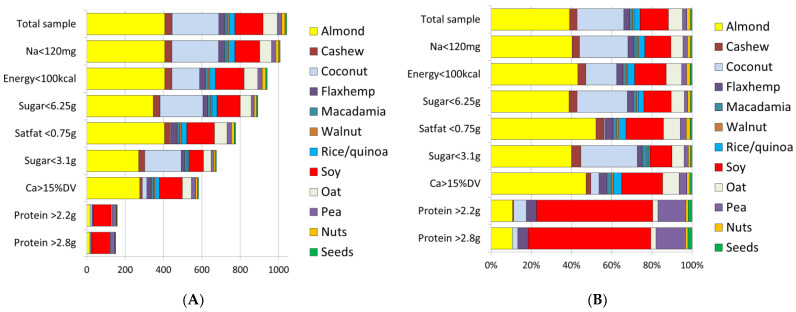
The number (**A**) and percent distribution of PB milk alternatives (**B**) that met the proposed nutrient standards by main ingredient.

Figure 3 shows how different PB milk alternatives met the proposed nutrient standards. Most PB beverages (other than coconut) contained <100 kcal/100 g and readily met the proposed energy standard. Virtually all products contained <120 mg/100 of sodium and so met the sodium standard. Reducing sodium to <60 mg still had a pass rate of 70% for PB milk alternatives and 100% for PB waters. Other than coconut milk, most PB beverages met the standard for saturated fat. Most PB milk alternatives met the proposed standard for total sugar for adults (<6.25 g/100 g); fewer met the standard for the “ideal” lower total sugar content (<3.1 g/100 g).

Protein was the limiting nutrient. Only 20% of PB milk alternatives met the proposed standard for protein (2.2 g/100 g) and only 15% met the more stringent standard of 2.8 g/100 g protein. The proposed composite nutrient density standards for PB milk alternatives were based on energy, protein, saturated fat, total sugars, calcium, vitamin A and vitamin D. Those standards were met by 117 products (11.2%). As shown in Figure 3, soy and pea products were most likely to meet the proposed protein standard. Only 80 out of 1042 PB (7.7%) milk alternatives met the more stringent “best of class” standards for protein (2.8 g/100 g), and for added sugar (<3.1 g/100 g).

### 3.4. Nutrient Density Assessed Using Alternative Nutrient Profiling Methods

**Figure 4:**
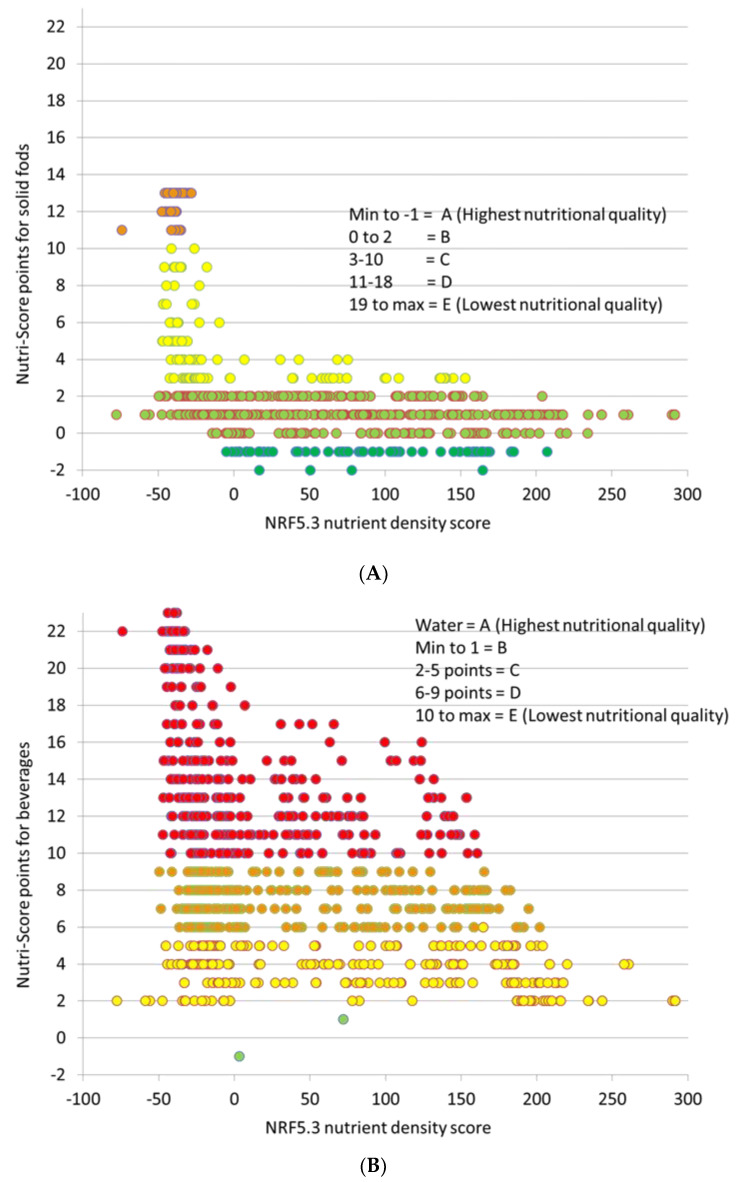
Plant-based beverages (milk alternatives and waters) rated using Nutri-Score for solid foods (**A**) and beverages (**B**). Nutri-Score points are plotted against NRF5.3 scores.

Two versions of Nutri-Score were used: one for beverages and one for solids. Based on current guidelines, all PB beverages are to be treated as solid foods by Nutri-Score. Using the Nutri-Score for solid foods (Figure 4A), most PB milk alternatives and PB waters scores within the 3–10 point range and some had Nutri-Score values <−1. Those products were rated A (highest nutritional quality) or B since their sugar content (mostly below 4.5 g/100 g) was not. There were no items that scored below D grade. The main ingredient of PB waters is sugar.

Using the Nutri-Score for beverages (Figure 4B), produced very different results. Only one product (low sugar coconut water with 3.8 g fiber) had a −1 point score and only 1 item had a +1 score. Both items received B letter grades. Some beverages received C grades. Most Nutri-Score point scores were 6 or above, corresponding to D and E grades. In other words, PB beverages previously graded as nutritious by Nutri-Score, were now assigned to the lowest nutritional quality groups. In general, lower scores (good) were given to almond, tree nut, and flax/hemp beverages followed by pea products. Coconut, rice, and oat beverages had higher scores (bad) and received lower grades.

The NRF5.3 nutrient density ratings plotted on the X axis of Figure 4 do not distinguish between beverages and solid foods and were the same in both cases. There was a negative correlation (r = −0.45) between Nutri-Score for beverages and the NRF5.3 nutrient density score. Many PB beverages with the same Nutri-Score values had very disparate NRF ratings. That is because the NRF5.3 score considers micronutrient fortification, whereas Nutri-Score does not.

Table 4 shows energy density (kcal/100 g) and mean nutrient density ratings by PB product type and by main ingredient. The PB are arranged in descending order of NRF5.3 scores for easier ranking. PB milk alternatives made from soy, almond and tree nut (macadamia, walnut) received the highest mean NRF5.3 scores, whereas coconut milks received the lowest. The main effect of product categories was significant (*p* < 0.001). However, given the small number of items in some categories, any differences between means ought to be interpreted with caution.

The two versions of Nutri-Score, one for solid foods and one for beverages, produced very different results. Lower Nutri-Score values denote higher nutritional value. Data in Table 4 show that the same PB beverages were rated by Nutri-Score as having a higher nutritional value when treated as solid foods, following current requirements. Paired t-test for all PB beverages was highly significant (t = −82.7; *p* < 0.001). Significant differences were obtained for every product category (<0.027 for quinoa; *p* < 0.001 for the rest). In all cases, PB beverages received higher Nutri-Score ratings when treated as solid foods.

The pass rate in the Choices system was highest for almond, tree nut, flax and hemp products. Coconut milks had a zero pass rate in the Choices system.

## 4. Discussion

PB milk alternatives are a rapidly growing market segment. Not all such products are equivalent to milk in terms of nutritional value [3,4,20]. In the US, milk is the main source of dietary calcium and vitamin D; provides multiple minerals critical to bone health, and contains potassium, iodine, riboflavin, and vitamin A [21]. At this time, only fortified soy milks are viewed by the USDA as substantially equivalent to milk.

Based on the present analyses, only 11% of PB milk alternatives in the USDA BFPDB met the previously proposed nutrient standards [12] that specified optimum energy, minimum protein content, maximum amounts for saturated fatand total sugars, as well as adequate fortification with calcium and vitamins A and D. Only 80 out of 1,042 (7.7%) PB milk alternatives met the more stringent standards for higher protein (2.8 g/100 g) and lower total sugars (<3.2 g/100 g). Most of those were fortified soy products.

Protein was the limiting nutrient; most of the PB milk alternatives–except pea and soy products–were low in protein. In many cases, beverage protein came from pea or soy concentrates or isolates. Increasing protein content of PB milk alternatives may present some technical challenges [22,23], not least that some of the protein concentrates or isolates contain sodium.

Even so, mean sodium content of PB milk alternatives was 47 mg/100 g; it was even lower for PB waters (20 mg/100 g). The proposed nutrient standards for sodium were <120 mg/100 g) and were met by virtually all PB milk alternatives. Even when the criterion was reduced to <60 mg/100 g, most PB beverages met the criterion. The present recommendation is to set the sodium standard for PB milk alternatives at <60 mg/100 g.

PB milk alternatives were frequently fortified with calcium, vitamin A, and vitamin D and less often with vitamin E, vitamin B12, and zinc. Some ingredient lists specifically mentioned vitamin mixes composed of calcium, vitamin A, and vitamin D. Not many PB milk alternatives were fortified with vitamin B2, but some had high levels of vitamin B12. Dairy milk contains calcium, vitamin B2 and vitamin B12 and is fortified with vitamin A and vitamin D in the US but not necessarily elsewhere.

Nutrient content of PB waters, another growing market segment, was also of interest, though there were no specific expectations, recommendations, or targets for this emerging product category. The main ingredient of PB waters was sugar, though present in amounts that were well below those in sugar sweetened beverages. Some PBB waters contained added vitamin C, and a few were fortified with multiple micronutrients.

The two Nutri-Score systems for beverages and for solid foods deserve a separate discussion. Based on the current French guidelines for Nutri-Score use, all PB beverages are to be treated as solid foods. The two versions of Nutri-Score—one for solid foods and one for beverages—differ in their scoring criteria, notably when it comes to energy and to added sugars. For solid foods, the initial threshold for energy is >335 kJ/100 g and that for total sugars is >4.5 g/100 g. As a result, most PB beverages passed the solid food nutrient quality test, which was very likely the intent. Treating PB beverages as solid foods leads to more favorable Nutri-Score ratings.

Treating PB beverages as beverages produces different results. In the beverage version of Nutri-Score, the “highest nutritional quality product” is plain water and only water is awarded the highest A grade. It bears reminding that water contains no calories and no nutrients. For the caloric beverages, the energy threshold is >30 kJ/100 g and the sugar threshold is >1.5 g/100 g. Those same PB beverages now score poorly, when assessed by Nutri-Score. The two versions of Nutri-Score, applied to the same PB beverages, produce very different results. By contrast, NRF5.3 values were the same for foods and beverages and were not affected by policy considerations.

Treating beverages as solid foods can lead to some unexpected situations. The USDA BFPDB lists products that are identified, described, and marketed as “plant-based milks” and “plant-based waters” [13]. The French government, copyright holder of Nutri-Score, requires all plant-based beverages to be treated as solid foods [19]. Presumably that includes plant-based waters. As a result, the current situation represents a duality of matter: PB waters that are treated as liquid beverages in the US are to be evaluated as solid foods in France. Unless the Nutri-Score is revised or exceptions are made, evaluating PB beverages correctly poses a challenge to some nutrient profiling systems.

Currently, Nutri-Score does not address protein quality [24] and does not consider product fortification. That can be viewed as a problem, given the high prevalence of micronutrient deficiencies among women and children worldwide [25]. As nutrient profiling methods are used to assess nutrient density of the global food supply, it is important to ensure that product innovations are correctly captured by nutrient density metrics and measures.

In summary, relatively few PB milk alternatives met the proposed nutrient standards for adequate protein. Proposed standards for sodium, energy and saturated fat were easier to meet Fortification with calcium and vitamins A and D was ubiquitous for some popular products (including soy and almond milks) but less common for others (cashew and tree nut). Many PB milk alternatives were sweetened; however, added sugars were present in amounts that were lower than those found in most sugar sweetened beverages. Reducing the added sugar standard from 6.35 g/100 g to <3.1 g/100 g reduced the number of PB milk alternatives that met the nutrient standards.

## 5. Conclusions

Protein was the limiting nutrient for the PB milk alternatives. Except tor soy and pea products, most PB milk alternatives had protein content below that found in milk. If this product category continues to be marketed as an alternative to milk, protein and protein quality remain important issues. No such expectations are attached to, e.g., plant-based waters. This emerging market category of plant beverages, including both PB milk alternatives and PB waters, would clearly benefit from repeated screenings for nutritional value. The Nutri-Score, which treats plant-based beverages as solid foods, is not particularly helpful in this regard. These challenges in assessing the nutritional value of new products will multiply as more PB beverages enter the global marketplace.

## Figures and Tables

**Table 1:** Selected ingredient lists for PB milk alternatives and PB waters.

ID	Declared Ingredients
496925	Organic almond milk (filtered water, organic almonds), organic cane sugar, organic locust bean gum, sea salt, natural flavors, sunflower lecithin, tricalcium phosphate, gellan gum, potassium citrate, organic vanilla extract, vitamin A palmitate, ergocalciferol (vitamin D2), DF-alpha-tocopherol acetate (vitamin E), cyanocobalamin (vitamin B12)
1097251	Flax milk (filtered water, cold pressed flax oil), pea protein isolate, cane sugar, tapioca starch, vanilla extract, natural flavors sunflower lecithin, sea salt, gellan gum, xanthan gum, vitamin A palmitate, vitamin D2, vitamin B12
411752	Cashew milk (filtered water, cashews), contains 2% or less of: almond butter, vitamin & mineral blend (incl. calcium carbonate, vitamin E acetate, vitamin A palmitate, vitamin D2), sea salt, locust bean gum, sunflower lecithin, natural flavor, gellan gum, ascorbic acid

**Table 2:** Proposed energy and nutrient content for PB milk alternatives.

	Proposed Nutrient Standards Per 100 g	
	Children (4–12 Years)	Adults (>12 Years)
Energy (kcal)	<85	<100
Protein (g)	>2.2	>2.2
Best of class protein (g)	>2.8	>2.8
Protein quality (PDCAAS)	>0.9	>0.8
Total/added/free sugars (g)	<5.3	<6.25
Best of class sugars (g)	<2.7	<3.1
Saturated Fat (g)	<0.75	<0.75
Sodium (mg)	<120	<120
Calcium (mg)	>15% DV/200 g serving	>15% DV 200 g serving
Riboflavin (B-2), (mg) Vitamin D (IU) Vitamin B-12 (mcg) Vitamin A, RAE (mcg)	>15% DV/200 g serving	>15% DV/200 g serving

DV daily value; IU international units; PDCAAS protein digestibility corrected amino acid score, RAE retinol activity equivalents.

**Table 3:** Energy and nutrient content per 100 g of PB milk alternatives and PB waters by product type and main ingredient. Data are means (SE). Significance tests (*p*-values) are for column means.

	N	Energy	Protein g	Total Sugars g	Total Fat g	Sat Fat g	Calcium mg	Sodium mg
		kcal/100 g	Mean (SE)	Mean (SE)	Mean (SE)	Mean (SE)	Mean (SE)	Mean (SE)
PB milk alternatives								
All	1042	49	1.14 (0.03)	2.80 (0.09)	3.21 (0.14)	1.86 (0.13)	97.6 (2.46)	47.1 (0.70)
Almond	407	27	0.71 (0.04)	2.58 (0.14)	1.37 (0.04)	0.06 (0.01)	128.6 (3.79)	62.4 (0.81)
Cashew	38	36	0.87 (0.11)	1.88 (0.30)	2.26 (0.25)	0.40 (0.09)	66.8 (12.64)	43.2 (4.30)
Coconut	243	95	0.70 (0.05)	2.12 (0.14)	8.66 (0.43)	7.43 (0.38)	23.7 (3.34)	26.5 (1.31)
Flax/hemp	30	37	1.46 (0.23)	3.00 (0.51)	1.64 (0.15)	0.11 (0.03)	104.9 (11.22)	41.3 (3.03)
Macadamia	15	27	0.31 (0.05)	1.29 (0.36)	2.29 (0.33)	0.42 (0.07)	166.6 (9.67)	44.0 (2.02)
Walnut	8	33	0.63 (0.14)	2.60 (0.93)	2.00 (0.57)	0.10 (0.07)	101.6 (25.67)	47.1 (7.70)
Rice	28	53	0.50 (0.07)	5.05 (0.51)	1.21 (0.13)	0.17 (0.09)	101.4 (10.68)	36.2 (1.77)
Quinoa	3	34	0.84 (0.00)	1.97 (1.13)	0.42 (0.00)	0.00 (0.00)	127.0 (0.00)	46.0 (0.00)
Soy	147	44	2.82 (0.05)	3.62 (0.19)	1.52 (0.03)	0.20 (0.01)	116.9 (4.94)	43.0 (1.37)
Oat	74	53	1.35 (0.08)	4.15 (0.39)	1.90 (0.15)	0.43 (0.11)	108.3 (7.79)	49.0 (1.75)
Pea	23	46	3.46 (0.08)	3.44 (0.58)	1.93 (0.10)	0.20 (0.02)	180.0 (9.00)	47.8 (3.27)
Nuts	18	41	1.01 (0.22)	3.51 (0.70)	2.29 (0.30)	0.42 (0.14)	68.3 (14.61)	47.3 (3.95)
Seeds/fruit	8	42	1.68 (0.51)	3.18 (1.43)	2.01 (0.27)	0.31 (0.11)	99.2 (17.20)	33.1 (6.36)
*p*-value		<0.001	<0.001	<0.001	<0.001	<0.001	<0.001	<0.001
PB waters								
All	550	23	0.07 (0.01)	4.60 (0.99)	0.08 (0.01)	0.05 (0.01)	13.1 (0.79)	20.72 (0.57
Coconut	464	23	0.08 (0.02)	4.72 (0.08)	0.09 (0.02)	0.06 (0.01)	13.9 (2.04)	23.38 (0.57)
Aloe	36	21	0.00 (0.00)	4.71 (0.60)	0.00 (0.00)	0.00 (0.00)	8.9 (2.04)	7.33 (1.21)
Tree waters	23	12	0.00 (0.00)	2.64 (0.30)	0.00 (0.00)	0.00 (0.00)	5.8 (0.81)	2.70 (1.18)
Fruit	15	23	0.09 (0.05)	4.87 (1.30)	0.00 (0.00)	0.00 (0.00)	5.1 (1.52)	8.87 (1.99)
Plain	12	12	0.03 (0.03)	3.29 (0.88)	0.10 (0.10)	0.00 (0.00)	18.5 (15.52)	7.50 (6.19)
*p*-value		<0.001	0.483	<0.001	0.242	0.456	<0.027	<0.001

**Table 4:** Mean energy and nutrient density scores for PB milk alternatives and PB waters by product type and main ingredient. Data are means (SEM). Significance tests (*p*-Values) are for column means.

PB Milk Alternatives		NRF5.3 *	Nutri-Score Beverage	Nutri-Score Solid Food	Choices
	N	Mean (SE)	Mean (SE)	Mean (SE)	Number Passing
All	1042	68.83(2.60)	9.20 (0.17)	2.32 (0.11)	421
Macadamia	15	141.48 (20.51)	5.27 (0.61)	1.07 (0.07)	14
Walnut	8	110.60 (26.89)	7.12 (1.06)	1.25 (0.16)	4
Soy	147	109.85 (5.20)	8.44 (0.27)	0.02 (0.06)	49
Almond	407	101.82 (3.83)	6.12 (0.18)	1.13 (0.04)	277
Flax/hemp	30	87.64 (12.83)	7.46 (0.81)	0.87 (0.22)	17
Pea	23	84.95 (10.51)	8.30 (0.96)	−0.70 (0.13)	7
Quinoa	3	62.40 (9.86)	7.00 (1.00)	1.00 (0.00)	2
Rice	28	60.40 (9.31)	11.36 (0.55)	1.64 (0.15)	2
Seeds/fruit	8	54.05 (22.24)	8.12 (1.86)	0.38 (0.56)	5
Nuts	18	53.42 (22.83)	9.17 (1.03)	1.28 (0.19)	6
Oat	74	48.40 (6.97)	10.44 (0.47)	1.19 (0.19)	22
Cashew	38	40.24 (13.07)	7.02 (0.59)	0.97 (0.10)	23
Coconut	243	−7.56 (3.97)	15.18 (0.37)	7.07 (0.29)	3
*p*-Value		<0.001	<0.001	<0.001	
PB waters					
All	550	−17.25(1.35)	7.13 (0.11)	1.60 (0.03)	52
Tree waters	23	51.10 (24.46)	4.47 (0.40)	1.22 (0.11)	13
Fruit	15	−6.47 (15.86)	6.73 (1.34)	1.80 (0.35)	5
Plain	12	−10.54 (22.34)	4.58 (1.08)	1.08 (0.26)	4
Aloe	36	−13.94 (9.46)	6.88 (0.76)	1.58 (0.14)	14
Coconut	464	−19.27 (1.14)	7.36 (0.10)	1.63 (0.03)	16
*p*-Value		<0.001	<0.001	0.001	

* NRF5.3 was calculated for items with ED > 10 kcal/100 g to avoid dividing by zero.

## Data Availability

The US Department of Agriculture Branded Food Products Database (BFPDB) is publicly available online at FoodData Central.

## Abbreviations

BFPDB	Branded Food Products Database
DV	Reference Daily Values
EU	European Union
FDA	Food and Drug Administration
FNDDS	Food and Nutrient Database for Dietary Studies
IU	International Units
LIM	Nutrients to limit sub-score
NRF	Nutrient-Rich Food index
PB	plant-based
QUID	Quantitative declaration of ingredients
USDA	US Department of Agriculture
